# Supplementary material for: Mechanistic Study on the Possibility of Converting Dissociated Oxygen into Formic Acid on χ-Fe5C2(510) for Resource Recovery in Fischer–Tropsch Synthesis
Source: Molecules. 2023 Dec 15;28(24):8117. doi: 10.3390/molecules28248117 (PMC10745954; doi:10.3390/molecules28248117)
Supplement: Supplementary file 1 [file molecules-28-08117-s001.zip › molecules-2652472-supplementary.pdf]

Supporting Information

Mechanistic study on the possibility of converting dissociated oxygen into formic acid on χ-Fe_5C_2(510) for resource recovery in Fischer-Tropsch synthesis

*Ning Ai[1,2,3], Changyi Lai[1,2], Wanpeng Hu[2], Qining Wang[3], Jie Ren[2]**

1 College of Chemical Engineering, Zhejiang University of Technology, Hangzhou 310014, China

2 College of Biological, Chemical Sciences and Engineering, Jiaxing University, Jiaxing, China.

3 National Demonstration Center for Experimental Chemistry and Chemical Engineering Education, Zhejiang University of Technology, Hangzhou 310014, China

An example of the different adsorption structures and representations of COOH species.

In Figure S1, it could be found that the configuration on the left hand side is different from the configuration on the right hand side. The configuration on the left is the product of CO_2 + H^1 = $COOH^1$ and is labeled "$COOH^1$". The configuration on the right is the product of CO_2 + H^2 = $COOH^2$ and is labeled "$COOH^2$". If other configurations of the same molecular formula exist, the numbers in the labeling will increase sequentially.

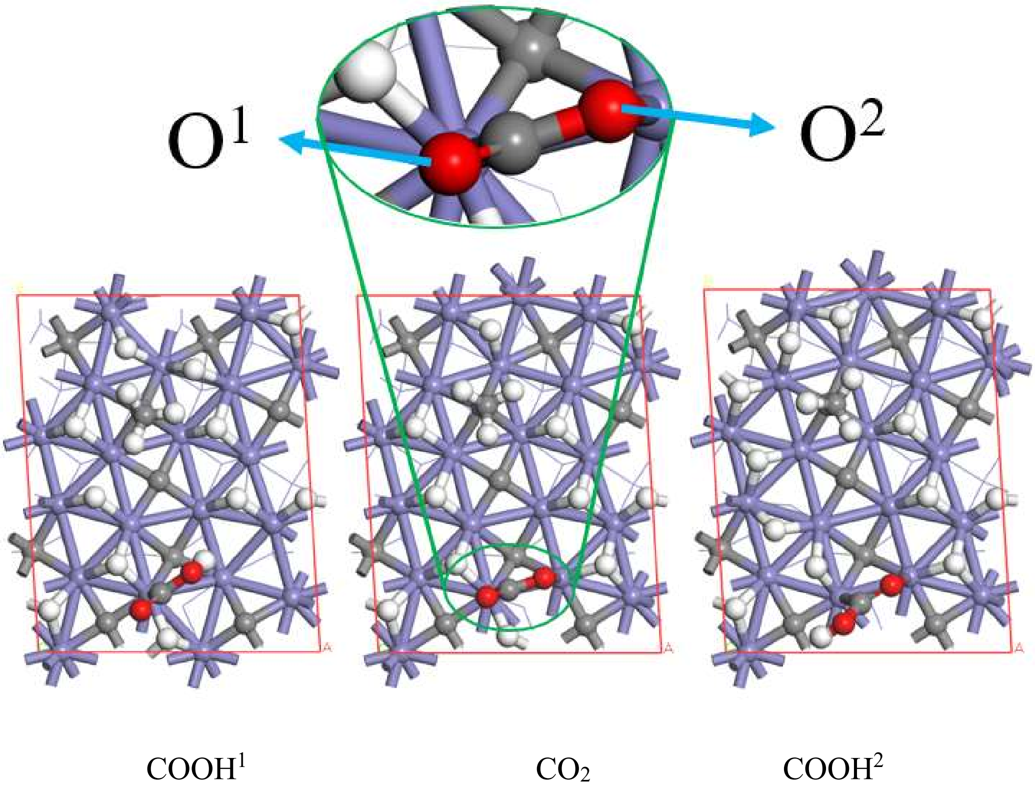

Figure S1 Conformations of COOH species: "$COOH^1$" as the product of the CO_2 + H^1 = $COOH^1$ reaction (left) and as the product of the CO_2 + H^2 = $COOH^2$ reaction (right).

An example of mutual transformation of different adsorption structures of the same species.

In Figure S2, the interconversion of HCOH, $HCOH^1$, and $HCOH^2$ is shown as an example of the interconversion of different adsorption structures of HCOH.

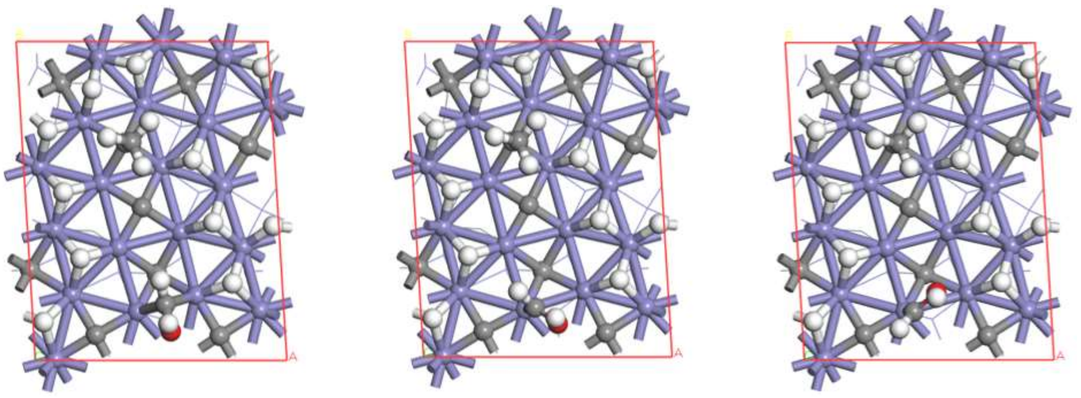

HCOH TS： HCOH→$HCOH^1$ $HCOH^1$

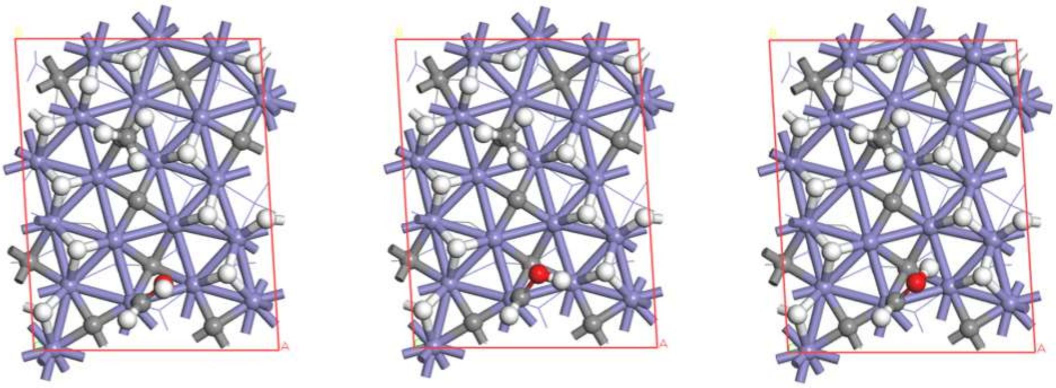

$HCOH^1$ TS： $HCOH^1$→$HCOH^2$ $HCOH^2$

Figure S2 Interconversion processes of different adsorption structures of HCOH.

The energy data concerning the dissociative O removal reaction and the initial, transition, and final states of the basic reaction

Table S1 Gibbs barrier energy (G_a, eV), Gibbs reaction energy (G_r, eV), and effective Gibbs barrier energy (G_{eff}, eV) for the basic reaction of the dissociative O removal reaction on χ-Fe_5C_2 (510)

Elementary reaction	G_a	G_r	G_{eff}
O+H=OH	0.74	-0.16	0.74
OH+H=H_2O	0.55	-0.31	0.55
CO+O=CO_2	0.85	0.28	0.85
CO+H^O=COH	1.79	0.66	1.79
COH+O=COOH	0.91	-0.69	0.91
COOH-1+H=HCOOH-2	0.63	0.40	0.64
COH+H^C=CHOH	0.66	0.41	0.66
CHOH+O=HCOOH-1	0.68	-0.20	0.68
CO+H^C=CHO	1.02	0.82	1.02
CHO+O=CHOO	0.73	-1.12	0.73
CHOO+O=HCOOH-3	0.58	0.38	0.58
CHO+H^O=CHOH	0.89	-0.02	0.89
CO+OH=COOH	0.84	0.75	0.84
COOH+H=HCOOH-5	0.19	0.05	0.19
CHO-O+H=CHO+OH	0.62	-0.42	0.62
CHO+OH=HCOOH-4	0.81	0.53	0.80
CHOH+H=H_2COH	0.24	-0.17	0.24
H_2COH+H=H_3COH	0.31	-0.39	0.31
CHO+H=H_2CO	0.19	0.15	0.19

H_2CO+H=H_2COH	0.54	-0.22	0.54
H_2COH+H=H_3COH	0.31	-0.39	0.31
H_2CO+H=H_3CO	0.24	-0.07	0.24
H_3CO+H=H_3COH	0.46	-0.76	0.46
H_2COH+H=H_3COH	0.31	-0.39	0.31
$COOH^1$=CO_2+H^1	0.55	-0.25	0.55
$COOH^2$=CO_2+H^2	1.01	0.57	1.01

The DFT data used in the kMC simulation in the Fischer-Tropsch reaction pathway

Table S2 The elementary reactions and corresponding kinetic parameters included in the kMC simulation about the Fischer-Tropsch reaction pathway on χ-Fe_5C_2(510)

Elementary reactions	k_{for}	$G_{a,f}$ (kcal/mol)	k_{res}	$G_{a,r}$ (kcal/mol)
H2(g)+ *→ H2*	1.18×10^{7}	-	1.00×10^{13}	5.53
H2*+2*→ 2H*	9.89×10^{12}	0.69	1.88×10^{13}	3.23
H*+ *→ *+ H*	1.28×10^{13}	3.92	1.28×10^{13}	
CO(g)+ *→CO*	3.18×10^{6}	-	1.00×10^{13}	32.98
CO*+*→*+ CO*	2.77×10^{13}	4.38	2.77×10^{13}	4.38
O+H→OH	4.1×10^{13}	17.15	1.42×10^{13}	20.84
OH+H→H_2O	5.85×10^{12}	12.75	1.16×10^{12}	20.03
CO+O→CO_2	2.67×10^{13}	19.67	2.67×10^{13}	13.25
CO+H^O→COH	1.90×10^{12}	41.24	1.18×10^{13}	25.95
COH+O→COOH	1.21×10^{13}	21.10	4.69×10^{12}	36.97
COOH→COOH-1	1.89×10^{13}	8.41	1.31×10^{13}	10.30
COOH-1+H→HCOOH-2	1.61×10^{13}	14.66	1.05×10^{12}	5.34
COH+H^C→CHOH	4.48×10^{12}	15.12	5.19×10^{12}	5.73
CHOH+O→HCOOH-1	1.00×10^{14}	15.78	2.71×10^{13}	20.40
CO+H^C→CHO	3.76×10^{12}	23.56	9.46×10^{12}	4.76
CHO+O→CHOO	5.90×10^{12}	16.76	1.74×10^{12}	42.67
CHOO+O→HCOOH-3	1.56×10^{14}	13.46	3.1×10^{13}	4.73
CHO+H^O→CHOH-1	1.28×10^{15}	20.63	1.35×10^{15}	21.19
CHOH-1-CHOH-2	3.23×10^{13}	24.26	2.4×10^{13}	9.96

CHOH-2-CHOH	2.40×10^{14}	3.34	2.92×10^{14}	7.74
CO+OH→COOH	1.51×10^{13}	19.29	9.59×10^{12}	2.03
COOH+H→HCOOH-5	1.49×10^{15}	4.32	1.86×10^{14}	3.06
CHO+O+H→CHO+OH	1.23×10^{13}	14.34	3.68×10^{15}	24.12
CHO+OH→HCOOH-4	5.56×10^{12}	18.62	5.60×10^{12}	6.34
CHOH+H→CH_2OH	1.45×10^{13}	5.61	5.27×10^{12}	9.53
CH_2OH+H→CH_3OH	2.75×10^{13}	7.11	5.66×10^{12}	16.18
CO+H→CHO	3.76×10^{12}	23.56	9.46×10^{12}	4.76
CHO+H→CH_2O	6.08×10^{13}	4.45	2.15×10^{13}	0.90
CH_2O+H→CH_2OH	2.71×10^{12}	12.40	3.97×10^{12}	17.50
CH_2OH+H→CH_3OH	2.75×10^{13}	7.11	5.66×10^{12}	16.19
CH_2O+H→H_3CO	6.36×10^{12}	5.44	3.9×10^{12}	7.09
H_3CO+H→CH_3OH	1.25×10^{13}	10.72	3.09×10^{12}	28.32
CHOH+H→CH2OH	1.45×10^{13}	5.61	5.27×10^{12}	9.53
CH_2OH+H→CH3OH	2.75×10^{13}	7.11	5.66×10^{12}	16.19
COOH→COOH-1	1.25×10^{13}	7.44	2.03×10^{13}	6.40
COOH-1→CO_2+H	6.29×10^{13}	12.65	5.1×10^{13}	18.47
COOH-2→CO_2+H	8.46×10^{12}	23.21	7.25×10^{12}	9.96
CH_2OH+H→CH_3OH	1.73×10^{13}	7.36	4.6×10^{12}	17.18

Figure S3 The initial, transition, and final states of the basic dissociative O removal reaction.

Elementary reaction	initial states	transition states	final states
O+H→OH	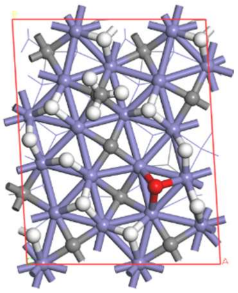	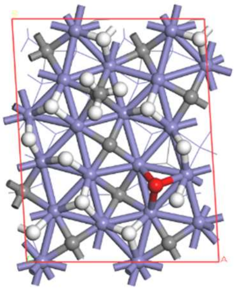	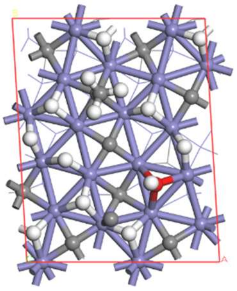
OH+H→HO	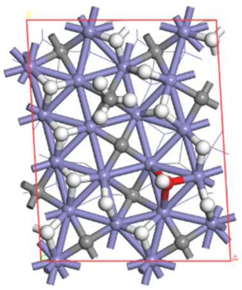	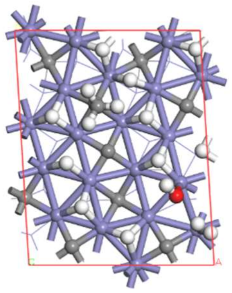	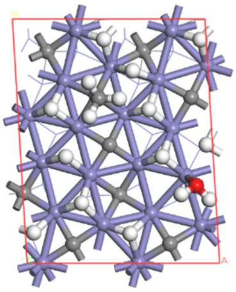
CO+O→CO_2	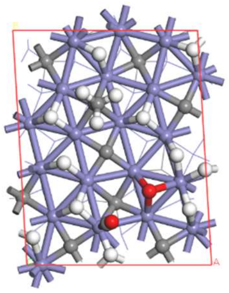	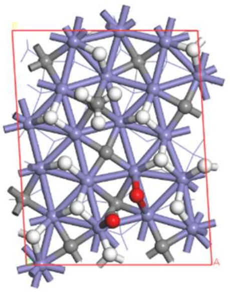	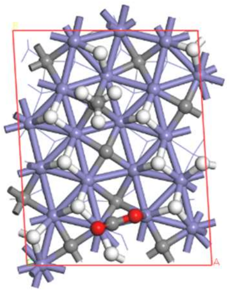
CO+H^C=CHO	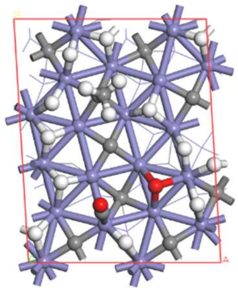	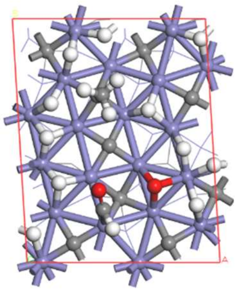	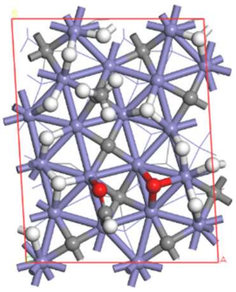

$CO+H^{O}=COH$

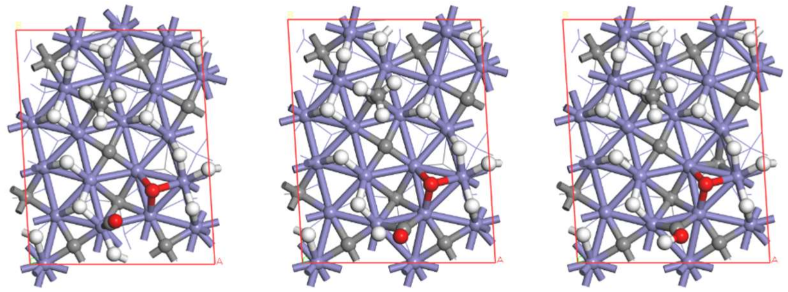

$COH+H^{C}=CHOH$

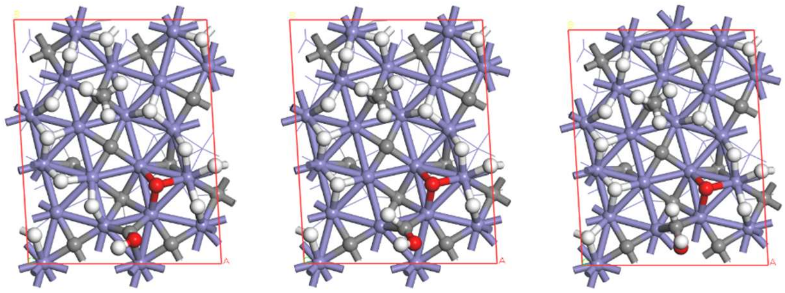

$CHO+H^{O}=CHOH$

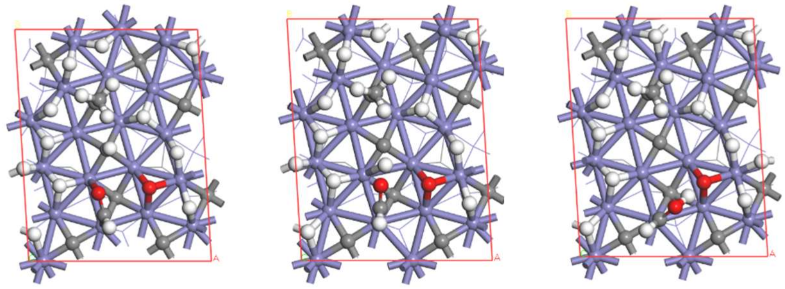

$CHO+O=CHOO$

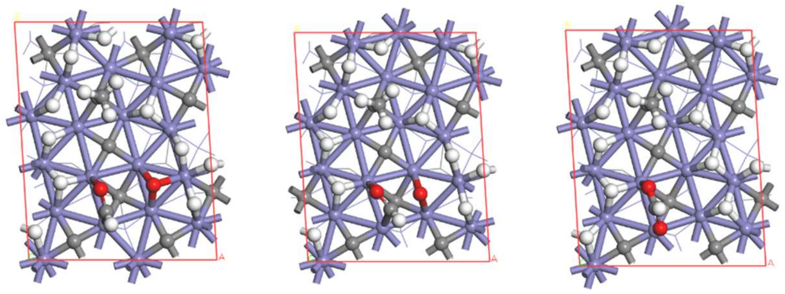

$COH+O=COOH$

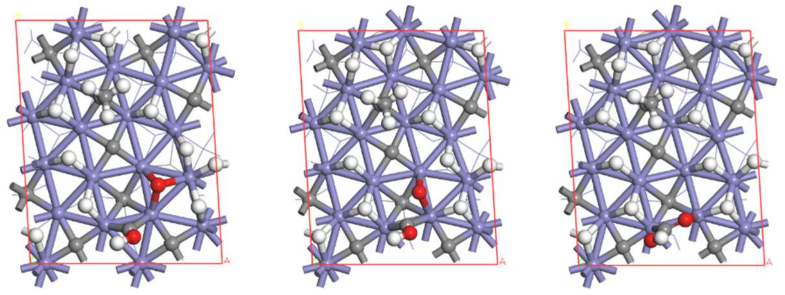

COOH+H=HCOOH-2

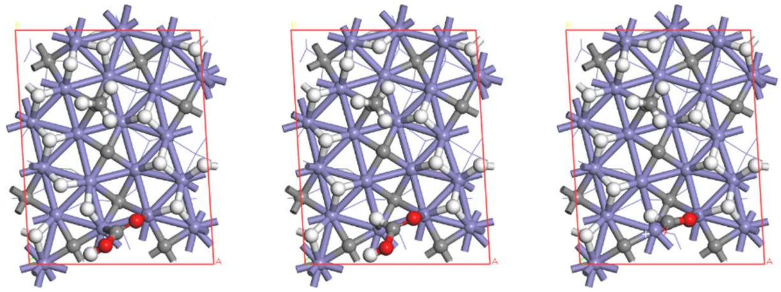

CHOH+O=HCOOH-1

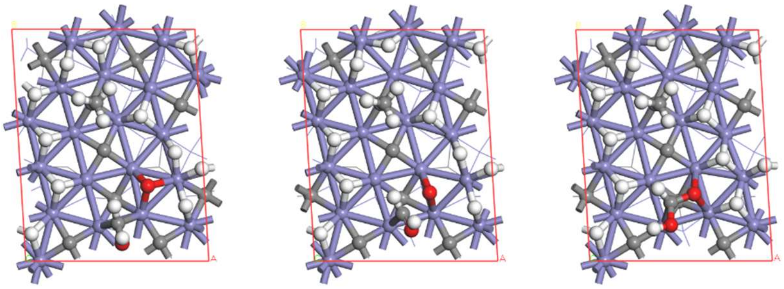

CO+OH=HCOOH-4

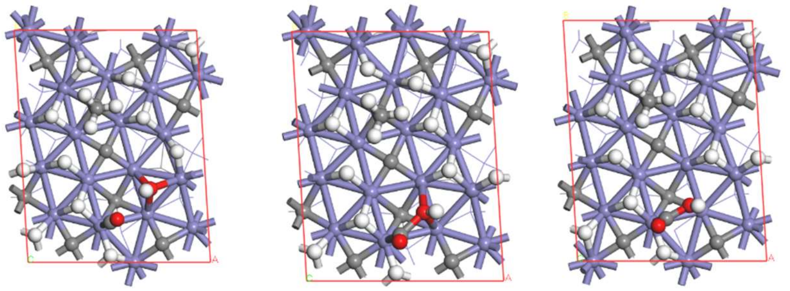

CHO+O+H=CHO+OH

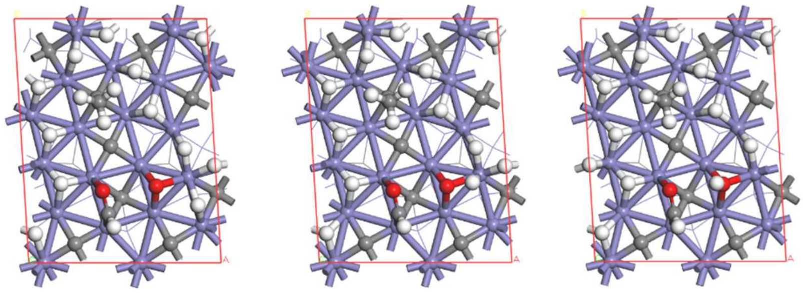

CHO+OH=HCOOH-5

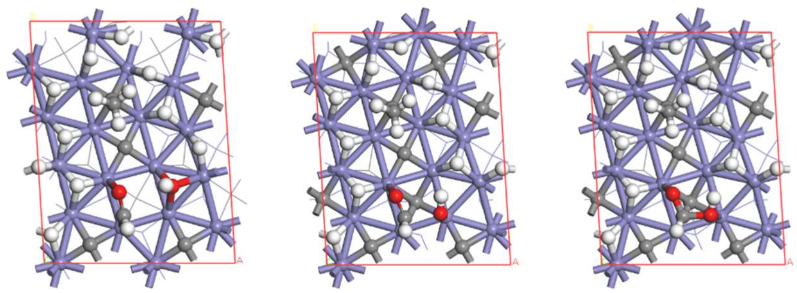

COOH+H=HCOOH

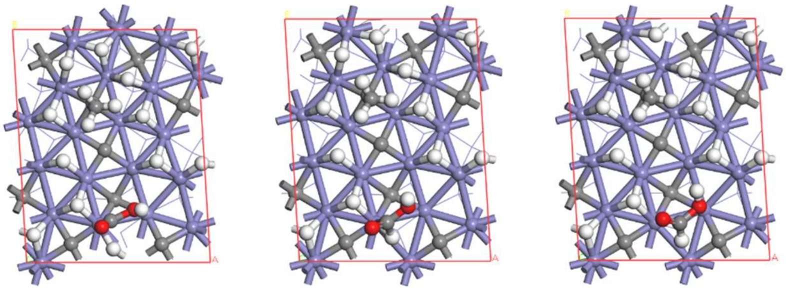

CHO+H=H_2CO

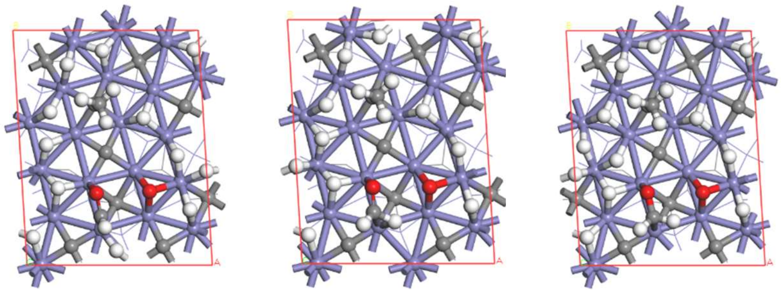

CHOH+H=CH_2OH

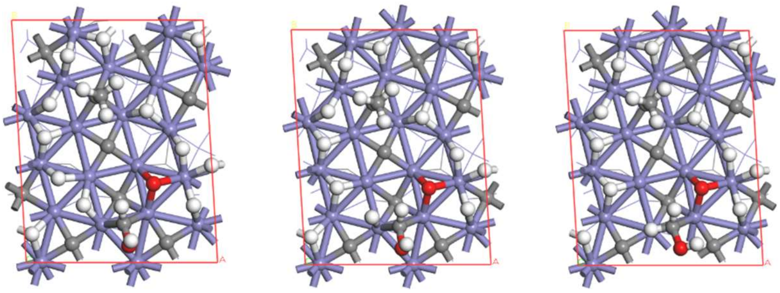

CH_2O+H=CH_2OH

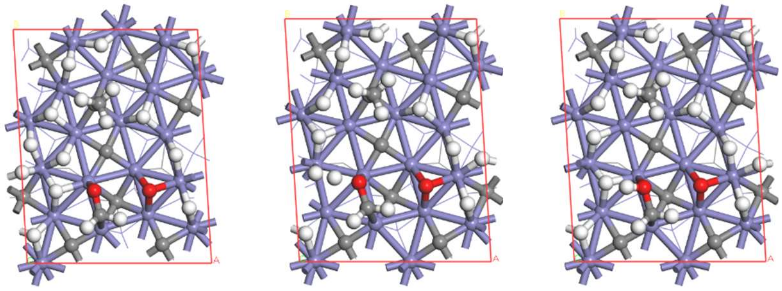

CH_2O+H=CH_3O

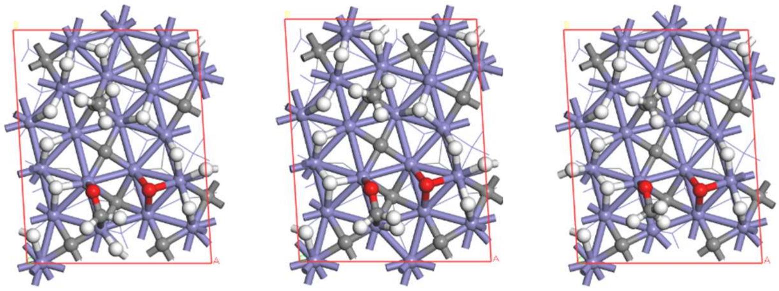

$CHOO+H=HCOOH$

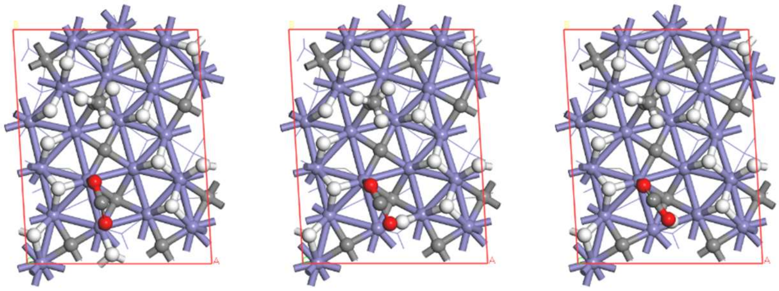

$CH_3O+H=CH_3OH$

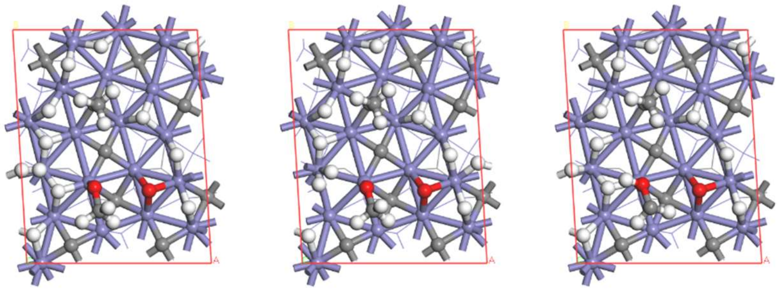

$CH_2OH+H=CH_3OH$

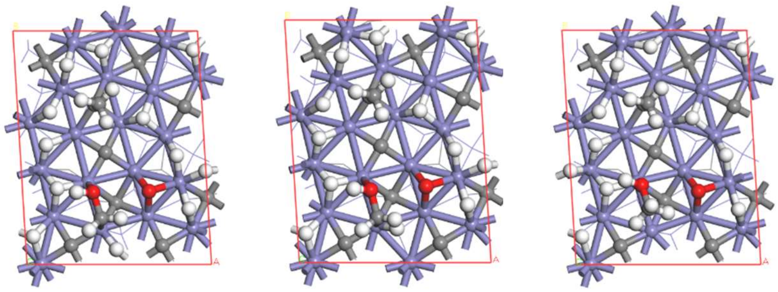

$CO_2+H=COOH$

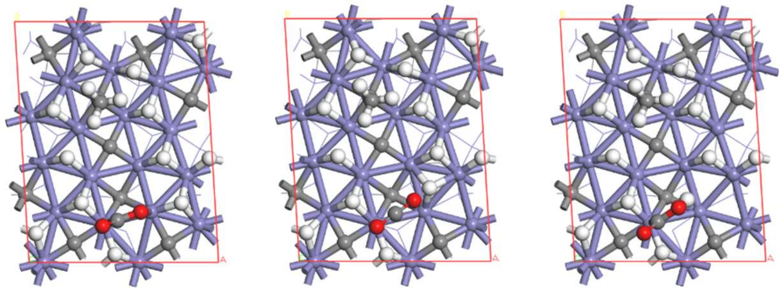

$CO_2+H=COOH$

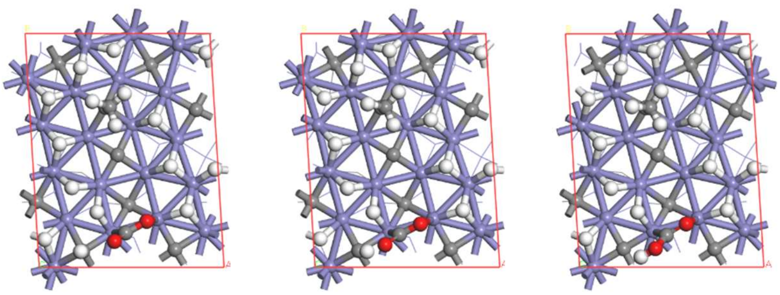

CHOH=CHOH[2]	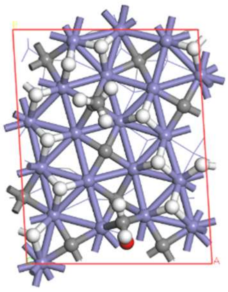	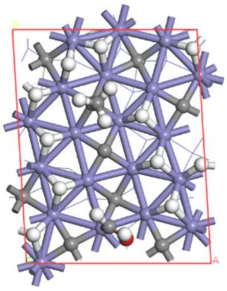	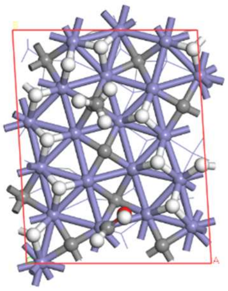
CHOH[1]=CHOH[2]	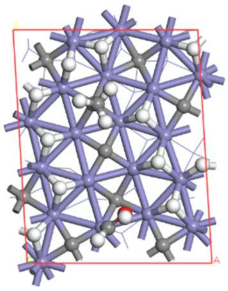	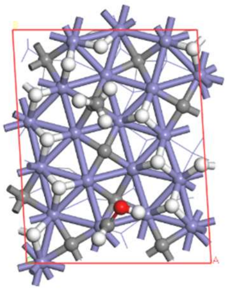	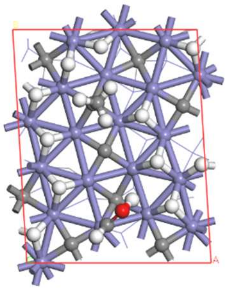
COOH=COOH[1]	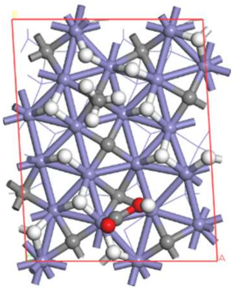	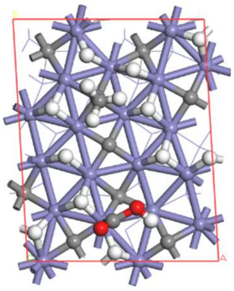	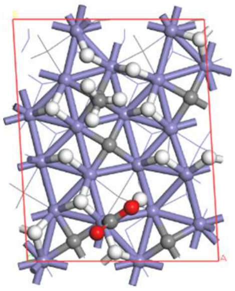
COOH[2]=COOH[3]	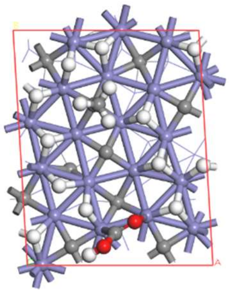	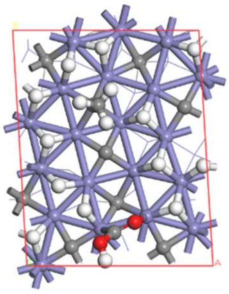	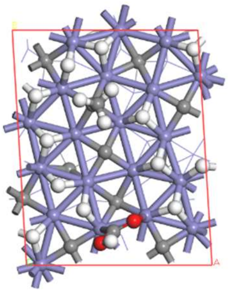

Tabel S3 Indicates the basic reaction corresponding to the transition state (TS) of Fig. 5

NO.	Elementary reactions	NO.	Elementary reactions
TS1	$CO+H^{C}=CHO$	TS2	$CO+O=CO_2$
TS3	O+H=OH	TS4	CHO-O+H=CHO+OH
TS5	$CO_2+H^{1}=COOH^{1}$	TS6	$CO_2+H^{2}=COOH^{2}$
TS7	OH+CO=COOH	TS8	CHOH+O=HCOOH-4
TS9	$COOH^{1}+H^{C}=HCOOH$-5	TS10	$COOH+H^{C}=HCOOH$-5
TS11	$COOH^{2}+H=HCOOH$-2		

Tabel S4 Indicates the basic reaction corresponding to the transition state (TS) of Fig. 6

NO.	Elementary reactions	NO.	Elementary reactions
TS1	$CO+H^{C}=CHO$	TS2	$CO+H^{O}=COH$
TS3	$CHO+H^{O}=CHOH$	TS4	COH+O =COOH
TS5	CHO+O=CHOO	TS6	$COH+H^{C}=CHOH$
TS7	CHOH+O=HCOOH-1	TS8	COOH+H=HCOOH-2
TS9	CHOO+H=HCOOH-3		

Tabel S5 Indicates the basic reaction corresponding to the transition state (TS) of Fig. 7

NO.	Elementary reactions	NO.	Elementary reactions
TS1	$CO+H^C=CHO$	TS2	$CO+O=CO_2$
TS3	O+H=OH	TS4	CHO+O =CHOO
TS5	$CO_2+H^1=COOH^1$	TS6	OH+CO=COOH
TS7	$COOH+H^C=HCOOH$-5	TS8	CHOO+H=HCOOH-3
TS9	$COOH^2+H=HCOOH$-2		

Tabel S6 Indicates the basic reaction corresponding to the transition state (TS) of Fig. 8

NO.	Elementary reactions	NO.	Elementary reactions
TS1	$CO+H^{O}=COH$	TS2	$CO+O=CO_2$
TS3	O+H=OH	TS4	O+H=OH
TS5	COH+O=COOH	TS6	OH+CO=COOH
TS7	$OH+H=H_2O$	TS8	$COOH1=CO_2+H$
TS9	$COOH=CO_2+H$		

Tabel S7 Indicates the basic reaction corresponding to the transition state (TS) of Fig. 9

NO.	Elementary reactions	NO.	Elementary reactions
TS1	$CO+H^{C}=CHO$	TS2	$CO+H^{O}=COH$
TS3	$CHO+H^{O}=CHOH$	TS4	COH+H =CHOH
TS5	$CHO+H=CH_2O$	TS6	$CH_2O+H=CH_2OH$
TS7	$CHOH+H=CH_2OH$	TS8	CH_2O +H=CH_3O
TS9	$CHOH+H=CH_2OH$	TS10	$CH_3O+H=CH_3OH$
TS11	CH_2OH+H= CH_3OH	TS12	CH_2OH+H= CH_3OH
TS13	CH_2OH+H= CH_3OH		CH_2OH+H= CH_3OH

Tabel S8 Indicates the basic reaction corresponding to the transition state (TS) of Fig. 10

NO.	Elementary reactions	NO.	Elementary reactions
TS1	$CO+O=CO_2$	TS2	$CO+O=CO_2$
TS3	O+H=OH	TS4	$CHO+H=CH_2O$
TS5	CO2+H=COOH	TS6	OH+CO=COOH
TS7	$OH+H=H_2O$	TS8	$CH_2O+H=CH_3O$
TS9	COOH+H=HCOOH	TS10	COOH+H=HCOOH
TS11	$CH_3O+H=CH_3OH$		
